# Cardiac proteostasis in obesity and cardiovascular disease

**DOI:** 10.1007/s00059-024-05233-6

**Published:** 2024-02-08

**Authors:** Joel Guerra, Leonardo Matta, Alexander Bartelt

**Affiliations:** 1 Institute for Cardiovascular Prevention (IPEK), Faculty of Medicine, Ludwig-Maximilians-Universität München, Max-Lebsche-Platz 30, 81377 Munich, Germany; 2 Institute for Diabetes and Cancer (IDC), Helmholtz Center Munich, German Research Center for Environmental Health, Neuherberg, Germany; 3 German Center for Cardiovascular Research, Partner Site Munich Heart Alliance, Munich, Germany; 4 German Center for Diabetes Research, Neuherberg, Germany

**Keywords:** Adipose tissue, Heart diseases, Autophagy, Endoplasmic reticulum, Proteasome, Fettgewebe, Herzerkrankungen, Autophagie, Endoplasmatisches Retikulum, Proteasom

## Abstract

Cardiovascular diseases (CVD) are closely linked to protein homeostasis (proteostasis) and its failure. Beside genetic mutations that impair cardiac protein quality control, obesity is a strong risk factor for heart disease. In obesity, adipose tissue becomes dysfunctional and impacts heart function and CVD progression by releasing cytokines that contribute to systemic insulin resistance and cardiovascular dysfunction. In addition, chronic inflammation and lipotoxicity compromise endoplasmic reticulum (ER) function, eliciting stress responses that overwhelm protein quality control beyond its capacity. Impairment of proteostasis—including dysfunction of the ubiquitin–proteasome system (UPS), autophagy, and the depletion of chaperones—is intricately linked to cardiomyocyte dysfunction. Interventions targeting UPS and autophagy pathways are new potential strategies for re-establishing protein homeostasis and improving heart function. Additionally, lifestyle modifications such as dietary interventions and exercise have been shown to promote cardiac proteostasis and overall metabolic health. The pursuit of future research dedicated to proteostasis and protein quality control represents a pioneering approach for enhancing cardiac health and addressing the complexities of obesity-related cardiac dysfunction.

## Abstract (de)

Kardiovaskuläre Erkrankungen stehen in enger Verbindung mit der Proteinhomöostase (Proteostase) und deren Kollaps. Neben genetischen Mutationen, welche die Qualitätssicherung von kardialen Proteinen beeinträchtigen, stellt Adipositas einen starken Risikofaktor für Herzerkrankungen dar. Bei Adipositas liegt eine Fehlfunktion des Fettgewebes vor, welche die Herzfunktion sowie den Verlauf von kardiovaskulären Erkrankungen beeinflusst, indem das Fettgewebe Zytokine freisetzt, die zur systemischen Insulinresistenz und zu kardiovaskulären Dysfunktionen beitragen. Darüber hinaus beeinträchtigen chronische Entzündung und Lipotoxizität die Funktion des endoplasmatischen Retikulums (ER) und lösen Stressreaktionen aus, welche die Qualitätskontrolle von Proteinen beeinträchtigen. Eine gestörte Proteostase, einschließlich der Dysfunktion des Ubiquitin-Proteasom-Systems (UPS), der Autophagie und des Verlusts von Chaperonen, ist eng mit der Dysfunktion von Kardiomyozyten verbunden. Interventionen, die mittels UPS- und Autophagie darauf abzielen, die Proteinhomöostase wiederherzustellen, sind potenziell neue Strategien, um die Herzfunktion zu verbessern. Anpassungen des Lebensstils, eine Ernährungsumstellung und verstärkte Bewegung haben sich als unterstützend für die kardiale Proteostase und die allgemeine Stoffwechselgesundheit erwiesen. Das Betreiben künftiger Forschung auf dem Gebiet der Proteostase und der Qualitätskontrolle von Proteinen stellt potenziell einen bahnbrechenden Ansatz zur Verbesserung der kardialen Gesundheit und zur Erfassung der Komplexität von durch Adipositas bedingten Herzkrankheiten dar.

Obesity is a disease of excess dysfunctional adipose tissue defined by a body mass index (BMI) of 30 kg/m^2^ or higher and is closely linked to a plethora of comorbidities including type 2 diabetes and cardiovascular disease (CVD). The impact of obesity on the cardiovascular system remains particularly concerning, as obesity induces metabolic and inflammatory changes, increasing the risk for life-threatening arrhythmias, atherosclerosis, and thrombosis [1].

At the molecular level, the concept of proteostasis—regulated homeostasis of protein metabolism—is emerging as a critical aspect in the context of cardiometabolic diseases. Cellular proteostasis is maintained by a tightly regulated network of molecular chaperones, proteases, and various quality control systems that collectively oversee protein translation, folding, and degradation [2]. Proteostasis is highly adaptive and indispensable for cellular function. However, in obesity, factors such as lipotoxicity, oxidative stress, and chronic inflammation impair this delicate balance, leading to the accumulation of misfolded and damaged proteins, particularly detrimental to cardiac structures and function [3]. New therapeutic approaches aiming at restoring proteostasis, including heat shock proteins and proteasome inhibitors, as well as non-pharmacological strategies such as dietary restriction and exercise, may have the potential to improve obesity-associated comorbidities. This review discusses the complex relationship between obesity, CVD, and proteostasis, highlighting the underlying basic biology, pathophysiological mechanisms, and therapeutic strategies.

## Obesity-induced dysfunction of the heart

Obesity, characterized by dysfunctional adipose tissue, is an endocrine disorder associated with a systemic inflammatory state and cardiovascular consequences. During excessive weight gain, adipose tissue undergoes a transformation from being a dedicated and safe lipid storage organ with a favorable endocrine profile to a major site of tissue inflammation and detrimental cytokine secretion. Adipocytes secrete various signaling molecules and cytokines such as leptin, adiponectin, and resistin, which normally maintain metabolic balance. However, in obesity, adipocytes become hypertrophic and the secretome changes, directly contributing to weight gain, metabolic stress, and systemic inflammation [4, 5]. The escalation of non-esterified fatty acid release from hypertrophic adipocytes further exacerbates metabolic stress by promoting systemic insulin resistance and ectopic lipid deposition in non-adipose tissues such as the liver, muscle, and particularly the heart, setting the stage for cardiovascular dysfunction [6]. In the cardiovascular system, the sequelae of obesity also manifest as altered hemodynamics, such as increased cardiac output, heightened systemic vascular resistance, and obesity-induced hypertension. This link between metabolic disruption and cardiovascular health is causal, as the direct impact of obesity on cardiac structure and function is evident, alongside the indirect effects mediated by obesity-associated comorbidities [1].

Lipotoxicity, a consequence of obesity, is characterized by the harmful accumulation of lipids within non-adipose tissues, including the heart, where they harm myocardial function. This lipotoxic environment prompts myocardial injury and triggers maladaptive cardiac remodeling, manifesting as fibrosis and cardiomyopathy, and compromising both diastolic and systolic heart functions [7]. Coupled with abnormal lipid profiles, obesity-related stress on cardiomyocytes, attributed in part to disturbed proteostasis, contributes to a deleterious cycle. Disrupted endoplasmic reticulum (ER) function induces a stress response that, when overwhelmed, leads to proteostasis imbalance, which is integral to maintaining cell viability and function. Given the robust epidemiological evidence linking obesity and CVD, a deeper understanding of the roles of ER stress and proteostasis in this interplay is warranted.

## Activation of the integrated stress response

Chronic metabolic overload and inflammatory stress disrupt ER function, an organelle pivotal in coordinating cellular metabolism and stress responses [8, 9]. The role of the ER in lipid and protein homeostasis is especially critical in cardiomyocytes, which demand precise coordination of protein synthesis and folding to sustain continuous cardiac function. Endoplasmic reticulum stress triggers the integrated stress response (ISR), including the unfolded protein response (UPR), ER-associated degradation (ERAD), and the ubiquitin-proteasome system (UPS). This preserves proteostasis, enhancing protein folding capabilities, regulating translation, and facilitating the targeted degradation of damaged proteins [10]. The role of the ER extends to regulate lipid synthesis and droplet formation, crucial for energy storage and utilization [11]. Furthermore, ER stress-induced lipotoxicity exacerbates systemic metabolic dysfunction, culminating in whole-body insulin resistance and, consequently, increased risk of developing CVD [7]. It is also noteworthy to mention that ER stress influences mitochondrial function through direct and indirect mechanisms. The ER and mitochondria are physically and functionally connected through structures known as mitochondria-associated ER membranes (MAMs). Disruptions in the integrity of MAMs lead to mitochondrial dysfunction, further worsening metabolic and cardiovascular outcomes [12].

Autophagy is another line of defense against ER stress in cardiomyocytes, orchestrating the degradation of protein aggregates to preserve cellular health. Autophagy is a multi-step cellular recycling process, initiated through the formation of a phagophore, a membrane that sequesters damaged proteins and organelles. These autophagosomes migrate and fuse with lysosomes, breaking down complex proteins into amino acids and other basic components [13]. The resulting macromolecular breakdown products are then recycled, ready to be repurposed for the synthesis of new proteins or to be utilized for energy production. The integrity of this system is especially vital in the context of cardiac physiology. During states of metabolic stress, such as those induced by obesity, the autophagic-lysosomal pathway is upregulated as a compensatory mechanism. It helps mitigate the accumulation of misfolded proteins that disrupt cardiac function, potentially leading to conditions such as cardiomyopathy and heart failure [14].

## Maladaptation of cardiac proteostasis

**Fig. 1:**
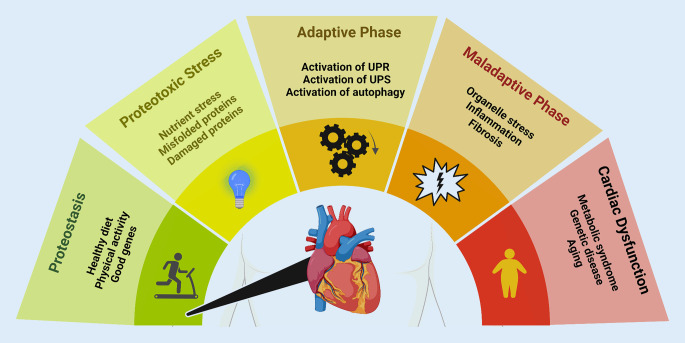
Cardiac proteostasis and its adaptation and dysfunction. Proteostasis describes a state of homeostatic protein production and degradation, usually found in healthy individuals, stimulated by healthy diets and physical activity. The occurrence of proteotoxic stress, e.g., presence of misfolded proteins and damaged proteins or simply caused by changes in proteome turnover are mitigated by the adaptive activation of the integrated stress response to restore homeostasis. If the proteotoxic stress persists, the cell enters a maladaptive phase characterized by organelle stress, inflammation, and fibrosis, which ultimately lead to cardiac dysfunction. Risk factors include aging, metabolic syndrome, and genetic predispositions. *UPR* unfolded protein response, *UPS* ubiquitin-proteasome system

Cardiomyocytes, with their specialized roles in electrical conduction and contraction, rely on the integrity of the proteome for optimal heart function. Due to the high metabolic demand of these cells, maintaining proteostasis presents unique challenges not commonly encountered by other cell types [15, 16]. Functional proteostasis is essential for a healthy heart, whereas proteome imbalances, such as misfolded proteins and aggregates, are hallmarks of cardiac disease (Fig. 1). The protein quality control systems, including the UPS and autophagy pathways, are critical in eliminating deleterious proteins and maintaining cellular integrity [17]. These systems are orchestrated by the UPR and ERAD. The UPR sensors, such as IRE‑1, PERK, and ATF6, respond to ER stress by activating adaptive pathways to restore proteostasis [18]. They also serve to rewire metabolism, as recently shown in a mouse model for Barth syndrome, in which activation of ATF4 compensated for defects in mitochondrial uptake of fatty acids to sustain energy production and antioxidation [19]. However, when overwhelmed, these sensors may trigger maladaptive responses, potentially leading to cardiac pathology as outlined here (Fig. 1).

The UPS is particularly sensitive to metabolic conditions such as obesity. Disruptions in UPS, exemplified by studies such as those involving PA28alpha (a proteasome activator) knockout mice, lead to the accumulation of polyubiquitinated proteins and desmin-related cardiomyopathy [20]. Unresolved chronic ER stress contributes to this by accumulating partially unfolded or misfolded proteins. Despite UPS activation in response to obesity and heart dysfunction, its capacity to mitigate proteotoxic stress may be inadequate or even maladaptive. Some of the detrimental outcomes of proteasome dysfunction seem to be linked to activation of ATF3 [21]. A key regulator of fine-tuning proteasome function and UPS is the NFE2-like bZIP transcription factor 1 (NFE2L1), a transcription factor that regulates the expression of genes coding of proteasome subunits and components [22, 23]. In the absence of stress, NFE2L1 is continuously degraded by a cascade of events involving deglycosylation by NGLY1 and proteolytic cleavage by DDI2 [24, 25]. However, during proteotoxic stress, for example, in the presence of chemical proteasome inhibitors or high levels of ubiquitylated proteins, NFE2L1 evades degradation and upregulates proteasome subunit production to counteract the stress. Deficiency of NFE2L1 has been linked to several physiologic and pathologic conditions, including compromised tissue regeneration and response to injury [26–28]. We and others have shown that loss of NFE2L1 is linked to ferroptosis, a cell death modality that involves lipid reactive oxygen species (ROS; [29]). Conversely, NFE2L1 overexpression enhances cardiac repair and protects against ischemia/reperfusion injury [28].

In humans, Predmore et al. reported reduced UPS activity in cardiac tissue samples obtained from patients with heart failure or hypertrophic cardiomyopathy, providing clinical evidence of proteostasis dysfunction in these heart diseases [30]. Furthermore, in a prospective study conducted by Makris et al., treatment with clinical proteasome inhibitors was associated with deteriorated cardiac function, reinforcing the significance of UPS in maintaining cardiac health. The study revealed left ventricular dysfunction and a deterioration of left atrial remodeling in these patients [31]. Heart diseases such as atrial fibrillation show impaired proteasome function, where sustained cardiomyocyte stress leads to dysfunctional autophagy and protease activity, resulting in contractile and electrophysiological deficits. This impaired proteostasis, observed in conditions of cardiac hypertrophy due to pressure overload, manifests as autophagic degradation and protein aggregation when these degradation systems are overwhelmed [32]. Moreover, a study of a population in southern Taiwan illustrated a notable decline in plasma ubiquitin and 20S proteasome levels in obese individuals compared to normal controls [33]. This intersection of UPR, ERAD dysfunction, and the resulting disruption of proteostasis are evident in progressive CVD and offer potential biomarkers for assessing obesity-related cardiovascular risk.

## Genetic impact on cardiac proteostasis

Cardiomyopathies are not only shaped by lifestyle factors but also by a spectrum of genetic mutations that directly influence cardiomyocytes proteostasis. Mutations in genes coding for valosin-containing protein (VCP) and BCL2-associated athanogene 3 (BAG3) primarily impact the proteasome. These *VCP* mutations lead to aberrant protein and RNA quality control, contributing to multisystem proteopathy with cardiac involvement [34]. The *BAG3* mutations are implicated in dilated cardiomyopathy, affecting the chaperone-assisted selective autophagy (CASA) complex, crucial for proteasomal degradation of mechanically strained proteins in cardiomyocytes [35].

Lysosomal-associated membrane protein‑2 (*LAMP2*) mutations result in Danon disease, characterized by lysosomal dysfunction and impaired autophagy, often presenting with hypertrophic cardiomyopathy [36]. The disruption of autophagic processes is a common theme in cardiomyopathies, highlighting the critical role of lysosomal degradation pathways in cardiac proteostasis. Dystrophin (*DMD*) gene mutations, causing Duchenne muscular dystrophy, lead to compromised sarcolemma integrity and secondary proteostasis imbalance, culminating in dilated cardiomyopathy [37]. Similarly, titin (*TTN*) truncating mutations disrupt sarcomere function due to the role of titin as a molecular spring, essential for the mechanical stability and proteostasis of cardiomyocytes [38]. Genetic variants in alpha-actinin‑2 (*ACTN2*) are associated with several forms of cardiomyopathy, featuring protein aggregation, hypertrophy, myofibrillar disarray, and activation of both the ubiquitin-proteasome system and the autophagy-lysosomal pathway [39]. Filamin C (*FLNC*) mutations lead to diverse cardiomyopathic outcomes. Aggregation-prone mutations in *FLNC* result in hypertrophic cardiomyopathy or myofibrillar myopathies, whereas mutations leading to haploinsufficiency are mainly associated with dilated cardiomyopathy and cardiac arrhythmias [40]. Also, these genetic factors delineate a complex landscape where the precise regulation of protein turnover is fundamental for cardiac function. Collectively, these clinical observations and experimental data converge to highlight the critical association between UPS and cardiomyopathies.

## Future directions in cardiovascular proteostasis management

Several therapeutic agents are in development that target protein quality control pathways, presenting new avenues for treating cardiomyopathies, including UPS and the autophagy pathway. While proteasome inhibition in cancer therapy is associated with cardiovascular side effects, there is potential for therapeutic applications in certain cardiac conditions, as restoring dystrophin complexes offers a new therapeutic strategy for muscular dystrophies such as Duchenne muscular dystrophy. The ability of the proteasome inhibitor bortezomib to mitigate progressive muscle degeneration could provide a significant benefit in preventing development of dilated cardiomyopathy and heart failure [41].

Traditional antimalarials, chloroquine and hydroxychloroquine, known for their anti-inflammatory effects, are being repurposed to inhibit autophagy, potentially mitigating autophagic vacuole accumulation in lysosomal disorders like Danon disease, where dysfunctional autophagy leads to pathological cardiac hypertrophy [42]. Conversely, spermidine has captured attention as an autophagy activator, with its selective induction of autophagy considered crucial for the clearance of aggregated proteins in cardiomyocytes, potentially improving cardiac outcomes in cardiomyopathies [43]. Additionally, molecules designed to enhance UPS function, such as USP14 inhibitors, are being developed to expedite the degradation of misfolded proteins, aiming to restore proteostasis [44].

Transthyretin stabilizers such as tafamidis and diflunisal present a novel approach to prevent the misfolding and aggregation of proteins implicated in amyloid cardiomyopathy [45]. Complementary to this, the chemical chaperone 4‑phenylbutyrate (4-PBA) is explored for its ability to enhance protein folding, potentially reducing the cardiac burden of proteotoxic stress [46]. Additionally, geranylgeranylacetone (GGA) is being studied for its capacity to induce heat shock proteins, a natural defense against misfolded proteins, offering protection in the context of cardiac proteostasis [47]. Tauroursodeoxycholic acid (TUDCA), with its chemical chaperone activity, is of interest for its potential to alleviate ER stress and modulate apoptotic pathways, which are critical in cardiomyocyte viability [48].

Complementing these pharmacological interventions, lifestyle modifications such as dietary restriction and regular exercise have been substantiated to beneficially modulate proteostasis. Dietary restriction, characterized by a controlled reduction in caloric intake that avoids malnutrition, orchestrates a cellular stress response pivotal for bolstering proteostasis. This adaptive response involves the upregulation of molecular chaperones that facilitate accurate protein folding, alongside the activation of proteolytic pathways crucial for the clearance of misfolded and potentially toxic proteins, thereby preventing protein aggregation and maintaining cellular homeostasis [49]. Complementarily, physical exercise exerts a cardioprotective effect by fostering adaptations of the cardiovascular system. One of the key mechanisms underlying this benefit is the activation of autophagy. Exercise-induced autophagy has been linked to enhanced cardiac function, attenuation of oxidative stress, and a deceleration of the age-associated decline in proteostasis, highlighting its role in the preservation of cardiac integrity [50]. Together, these strategies illustrate a concerted, pathway-oriented effort to combat cardiac diseases, spotlighting the integral role of protein quality control in the pathogenesis and treatment of cardiomyopathies.

## Conclusion

Obesity-induced heart disease represents a spectrum of immunometabolic cellular changes linked to increased risk for cardiovascular disease (CVD). This underscores the need for a deeper understanding of the nuanced mechanisms contributing to obesity-related cardiovascular complications, especially as the prevalence of obesity rises globally among aging populations. At the heart of these mechanisms lies proteostasis, the delicate equilibrium of protein synthesis, folding, and degradation, which is essential for cardiac function. Perturbations in this system are increasingly recognized as precipitating factors in the development and progression of cardiomyopathies, not only tied to obesity but as a generalizable key element of heart biology. In light of these findings, it will be relevant to intensify research into novel interventions that bolster proteostasis. As we advance our understanding of proteostasis in the context of cardiac health, the potential to devise more effective treatments for obesity-related CVD becomes increasingly tangible.

## Declarations

For this article no studies with human participants or animals were performed by any of the authors. All studies mentioned were in accordance with the ethical standards indicated in each case.
